# Dose-Dependent Effects of GLD-2 and GLD-1 on Germline Differentiation and Dedifferentiation in the Absence of PUF-8

**DOI:** 10.3389/fcell.2020.00005

**Published:** 2020-01-24

**Authors:** Youngyong Park, Samuel O’Rourke, Faten A. Taki, Mohammad A. Alfhili, Myon Hee Lee

**Affiliations:** ^1^Department of Internal Medicine, Division of Hematology/Oncology, Brody School of Medicine at East Carolina University, Greenville, NC, United States; ^2^Department of Pharmacology, Weill Cornell Medical College, New York, NY, United States; ^3^Department of Clinical Laboratory Sciences, College of Applied Medical Sciences, King Saud University, Riyadh, Saudi Arabia

**Keywords:** PUF-8, GLD-2, *C. elegans*, differentiation, dedifferentiation

## Abstract

PUMILIO/FBF (PUF) proteins have a conserved function in stem cell regulation. *Caenorhabditis elegans* PUF-8 protein inhibits the translation of target mRNAs by interacting with PUF binding element (PBE) in the 3′ untranslated region (3′ UTR). In this work, an *in silico* analysis has identified *gld-2* [a poly(A) polymerase] as a putative PUF-8 target. Biochemical and reporter analyses showed that PUF-8 specifically binds to a PBE in *gld-2* 3′ UTR and represses a GFP reporter gene carrying *gld-2* 3′ UTR in the *C. elegans* mitotic germ cells. GLD-2 enhances meiotic entry at least in part by activating GLD-1 (a KH motif-containing RNA-binding protein). Our genetic analyses also demonstrated that heterozygous *gld-2(+/−) gld-1(+/−)* genes in the absence of PUF-8 are competent for meiotic entry (early differentiation), but haplo-insufficient for the meiotic division (terminal differentiation) of spermatocytes. Indeed, the arrested spermatocytes return to mitotic cells via dedifferentiation, which results in germline tumors. Since these regulators are broadly conserved, we thus suggest that similar molecular mechanisms may control differentiation, dedifferentiation, and tumorigenesis in other organisms, including humans.

## Introduction

During development, stem cells must make a number of major fate decisions – the initial decision to either proliferate or differentiate, followed by whether to remain in a differentiating state or revert to being undifferentiated as occurs in regeneration or tumorigenesis. A regulatory network controlling these decisions is vital to the development of all multicellular organisms, including humans. Aberrant regulation can result in either loss of a specific cell type or uncontrolled cell proliferation, leading to tumors. To date, significant progress has been made in stem cell differentiation using multiple model systems. Nevertheless, our understanding of how differentiating cells maintain their state and how they are directed to a desired cell type remains largely deficient.

**FIGURE 1:**
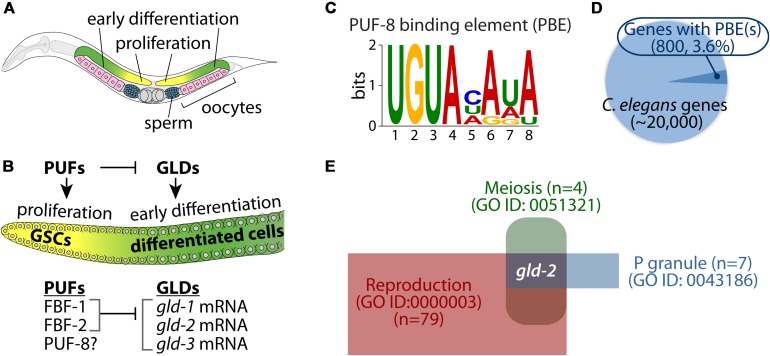
*Caenorhabditis elegans* germ line and PUF-8 RNA-binding protein. **(A)** Schematic of an adult *C. elegans* hermaphrodite gonad. Cells at the distal end of the germline include germline stem cells (GSCs) and proliferative cells (yellow). As cells move proximally, they enter meiosis (green) and differentiate into either sperm (blue) or oocytes (pink). **(B)** Key RNA-binding proteins that control a balance between proliferation and differentiation. PUFs proteins (e.g., FBF-1/2) promote germ cell proliferation by inhibiting GLDs (e.g., GLD-1/2/3)-mediated germline differentiation (Kimble and Crittenden, 2002). However, PUF-8 controls both proliferation and differentiation, depending on genetic context (Datla et al., 2014). **(C)** Consensus sequence of PUF-8 binding element (PBE). **(D)** Pie chart of potential PUF-8 target genes (800, 3.6%) that contain at least one PBE. **(E)** Identification of *gld-2* as a potential PUF-8 target mRNA involved in three gene ontology (GO) terms.

It is widely recognized that *Caenorhabditis elegans* germline provides an attractive model system for studying the differentiation of stem cells *in vivo*. Specifically, *C. elegans* germline is organized in a simple linear fashion that progresses from germline stem cells (GSCs) at one end to maturing gametes at the other (Figure 1A). Germ cells progress from GSCs at the distal end, through meiotic prophase as they move proximally to become differentiated gametes (sperm and oocytes) at the proximal end (Figure 1A). This developmental process requires a battery of RNA regulators (Kimble and Crittenden, 2002; Figure 1B). One of the well-studied families of RNA regulators important for germ cell development is the PUF family of RNA-binding proteins. The PUF protein binds a specific regulatory element in its target mRNA 3′ untranslated regions (3′ UTRs) and inhibits the expression of its target mRNAs by recruiting translational repressor complexes (Wickens et al., 2002). These include cytoplasmic Ccr4p-Pop2p-Not deadenylase complex (Goldstrohm et al., 2007) and Ago-eEF1A translational complex (Friend et al., 2012).

The *C. elegans* has multiple PUF proteins with specialized roles in germline and somatic tissues. Of those, three PUF proteins (FBF-1, FBF-2, and PUF-8) are highly expressed in the *C. elegans* germline and have critical roles in the maintenance of GSCs and mitotic germ cell fate. Specifically, FBF-1 and FBF-2 (collectively FBF) proteins are 95% identical, and they maintain GSCs by repressing the expression of genes that are associated with germline differentiation, including *gld-1* (a KH-motif containing RNA-binding protein) (Crittenden et al., 2002), *gld-2* [a poly(A) polymerase] (Millonigg et al., 2014), and *gld-3* (a bicaudal-C homolog) (Eckmann et al., 2004; Figure 1B). Another *C. elegans* PUF protein, PUF-8 (a PUF with a striking similarity to human PUMILIO) controls multiple cellular processes such as proliferation, differentiation, and the sperm-oocyte decision, depending on the genetic context (Datla et al., 2014). It has also been reported that PUF-8 acts as a tumor suppressor by inhibiting GLP-1 (one of two *C. elegans* Notch receptors) (Racher and Hansen, 2012) and MPK-1 (*C. elegans* ERK/MAPK homolog) signaling pathways (Cha et al., 2012). Notably, many cancer cell lines circumvent PUF-mediated regulation of E2F transcription factor, a known oncogene that is dysregulated or overexpressed in cancer (Miles et al., 2012). Therefore, elucidating the biological function of PUF-8 and its target genes will provide insights into the proliferation and differentiation of stem cells as well as contribute to our understanding of tumorigenesis in other animals, including humans.

In this study, we have identified *gld-2* as a direct target of PUF-8 repression in the *C. elegans* germline. Our genetic functional analyses showed that GLD-2 exhibits distinct functions depending on gene dosage in the absence of PUF-8. Under physiological conditions, two copies (+/+) of wild-type *gld-2* gene promote the differentiation of GSCs by working with GLD-1. One dose (+/−) of wild-type *gld-1* and *gld-2* genes, however, in the absence of PUF-8 promotes the formation of germline tumors via the regression of spermatocytes into mitotic cells (dedifferentiation) by activating MPK-1. Collectively, these findings suggest that a regulatory network involving PUF-8 and its repressing target, GLD-2, can promote either differentiation or dedifferentiation of germ cells through GLD-1 and MPK-1, depending on gene dosage.

## Results

### *In silico* Approach

*Caenorhabditis elegans* PUF-8 is a sequence-specific RNA-binding protein (Opperman et al., 2005). PUF-8 specifically binds to a regulatory element, termed the “PUF-8 binding element (PBE)” in target mRNA 3′ UTRs (Opperman et al., 2005; Figure 1C). PUF-8 is most similar to human PUM2 (Wickens et al., 2002; Subramaniam and Seydoux, 2003). Human PUM2 protein also binds to the same binding sequences, called Nanos Response Element (NRE), in target mRNA 3′ UTRs (Galgano et al., 2008; Bohn et al., 2018). Increasing evidence has shown that many genes with conserved PBEs were validated as PUF targets *in vitro* and *in vivo* (Prasad et al., 2016; Bohn et al., 2018). We thus performed an *in silico* approach to identify potential PUF-8 target genes from *C. elegans* whole genomes. Briefly, *C. elegans* 3′ UTR sequences were obtained from BioMart, and we identified 800 genes (3.6%) harboring at least one PBE in their 3′ UTRs (Figure 1D and Supplementary Table S1). To investigate functional themes among the 800 potential PUF-8 targets, we used DAVID and PANTHER tools (Dennis et al., 2003; Huang da et al., 2009) to look for enriched categories of biological processes, as defined in the gene ontology (GO) database (Supplementary Table S2). The most enriched GO terms were related to cellular processes, developmental processes, post-translational modification, and cell cycle. Of those, we have focused on P granule-associated proteins (GO ID: 0043186) that function in reproduction (GO ID: 0000003) and meiosis (GO ID: 0051321) (Figure 1E and Supplementary Table S3). Notably, *gld-2* gene was nominated for a putative PUF-8 target included in our selected GO terms (Figure 1E). The *C. elegans gld-2* gene encodes a poly(A) polymerase that is critical for the germline differentiation (Kadyk and Kimble, 1998; Wang et al., 2002).

### PUF-8 Binds a PBE in *gld-2* 3′ UTR

**FIGURE 2:**
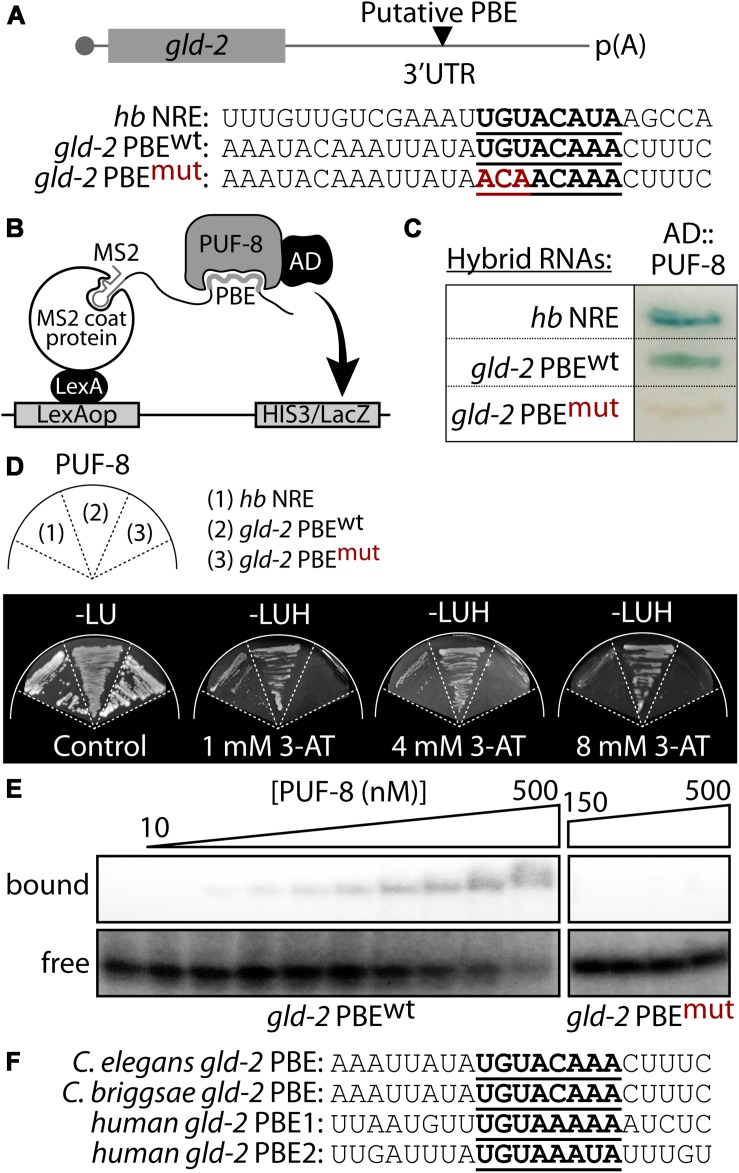
PUF-8 binds specifically to a PBE in *gld-2* 3′ UTR. **(A)** A putative PBE in *gld-2* 3′ UTR. Nucleotide sequences of a predicted PBE (see bold letters). Wild-type sequence is followed by its mutant, in which UGU is replaced by ACA. *hunchback (hb)* NRE (Nanos Response Element) served as a positive control for PUF-8 binding. **(B)** Schematic of yeast three-hybrid assay. **(C)** Three-hybrid interactions assayed by β-galactosidase activity. **(D)** *HIS3* reporter activation. Growth was monitored on media lacking histidine and containing different concentration of *HIS3* competitor 3-AT. **(E)** Gel retardation assay. Purified PUF-8 binds *gld-2* PBE^wt^, but does not bind *gld-2* PBE^mut^ with an altered consensus as detailed in panel **(A)**. **(F)** Sequence alignment of *gld-2* PBEs from *C. elegans*, *Caenorhabditis briggsae*, and *human*s.

The *gld-2* 3′ UTR (1,099 bp) possess one highly conserved PBE (Figure 2A). To assess PUF-8 binding to the predicted *gld*-2 PBE, we used yeast three-hybrid assay as previously described (Hook et al., 2005; Figure 2B). The yeast three-hybrid system is a useful tool in analyzing protein-RNA interaction. As previously described (Bernstein et al., 2002), a chimeric protein containing both a DNA- and RNA-binding domain tethers RNA to the promoter of a reporter gene. This protein consists of a LexA/MS2 coat protein fusion. A hybrid RNA binds to the MS2 coat protein via tandem MS2-binding sites. A hybrid RNA carrying the query sequence can bridge the LexA-MS2 and GAL4AD-PUF-8 hybrid proteins if PUF-8 binds, which is not possible if PUF-8 fails to bind. Yeast three-hybrid interactions were monitored by production of β-galactosidase from a *lacZ* reporter. The results indicate that PUF-8 interacts specifically with wild-type *gld-2* PBE (*gld-2* PBE^wt^) and a positive control, *hunchback* NRE (*hb* NRE), in yeast three-hybrid assays (Figure 2C and Supplementary Figure S1). By contrast, mutant *gld-2* PBE (*gld-2* PBE^mut^) with an altered consensus (UGU changed to ACA) did not interact with PUF-8 (Figure 2C). The strength of the PUF-8-*gld-2* PBE interaction was determined by the expression of a HIS3 reporter with upstream LexA operators. HIS3 expression confers growth on media without histidine and with 3-amino 1,2,3-triazol (3-AT) that is a competitive inhibitor of the HIS3-gene product. Notably, no significant growth was observed for *gld-2* PBE^mut^ strain, but significant growth of the positive control, *hb* NRE, and *gld-2* PBE^wt^ strains was detected at a 3-AT concentration of 4 and 8 mM, respectively (Figure 2D). This result indicates that an interaction between PUF-8 and *gld-2* PBE^wt^ is strong. A direct interaction between PUF-8 and *gld-2* PBE^wt^ was determined by gel retardation assay (Figure 2E and Supplementary Figure S2). Wild-type *gld-2* PBE^wt^ bound specifically to purified PUF-8 in gel shifts, but *gld-2* PBE^mut^ with an altered consensus did not interact with PUF-8. The apparent Kd value for PBE is about 110 nM. These results indicate that PUF-8 specifically binds to a PBE^wt^ within *gld-2* 3′ UTR. We also asked if a *gld-2* PBE is conserved in another nematode species, *Caenorhabditis briggsae gld-2* 3′ UTR, and in human Gld2 3′ UTR. Intriguingly, the *C. briggsae gld-2* mRNA has one conserved PBE and the human Gld2 mRNA has two conserved NREs in their 3′ UTRs (Figure 2F). Since these genes are highly conserved, we speculate that human PUM2 might also bind human Gld2 NRE, paving the way for an area of inquiry that warrants further pursuit.

### PUF-8 Represses *gld-2* mRNA Expression *in vivo*

**FIGURE 3:**
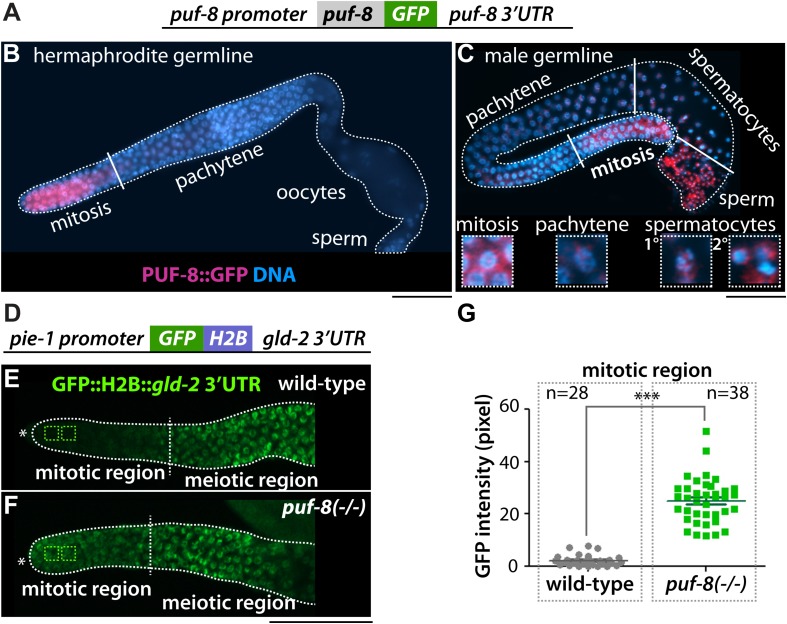
PUF-8 represses the expression of *gld-2* in the distal germline. **(A)** The design of the PUF-8:GFP fusion. **(B,C)** PUF-8:GFP expression in adult hermaphrodite and male germlines. **(D)** The design of the *gld-2* 3′ UTR fusion. The *pie-1* promoter is permissive for expression in all germ cells. **(E,F)** Staining of dissected gonads with anti-GFP antibody. The expression of *GFP:H2B:gld-2* 3′ UTR in the distal germlines of wild-type and *puf-8(–/–)* mutant worms. ^∗^Distal end of gonads. Scale bars, 50 μm. **(G)** Quantitation of GFP expression in wild-type and *puf-8*(−/−) mutant distal mitotic region [see squares in panels **(E,F)**].

PUF-8 expression was determined using a transgenic worm expressing a *puf-8 (promoter):GFP:puf-8 cDNA:puf-8 3′ UTR* transgene (Ariz et al., 2009; Racher and Hansen, 2012; Figure 3A). In adult hermaphrodite germline, the *GFP:puf-8* was expressed in the distal mitotic germ cells (Ariz et al., 2009; Racher and Hansen, 2012; Figure 3B). However, in adult male germline, the *GFP:puf-8* was expressed in distal mitotic germ cells, spermatocytes, and sperm (Figure 3C). Similar expression pattern was also observed in L4 staged spermatogenic hermaphrodite germline (Supplementary Figure S3). To test if PUF-8 might repress *gld-2* expression in the distal mitotic germ cells, we have also generated a transgenic worm expressing a *GFP:gld-2* 3′ UTR transgene in the germline. 3′ UTRs control protein expression temporally and spatially. We fused a GFP reporter to the *gld-2* 3′ UTR that contains a PBE and poly(A) signal sequences (Figure 3D). GFP expression in the germline was visualized by staining dissected adult hermaphrodite gonads with an anti-GFP polyclonal antibody and DAPI. The *GFP:gld-2* 3′ UTR was expressed at a low level in the distal mitotic germ cells, but was abundant in the differentiating meiotic cells [increased in the transition zone and became abundant in the pachytene (Figure 3E)] and oogenic cells (data not shown). To ask whether PUF-8 inhibits *gld-2* expression via its 3′ UTR, we introduced *puf-8(q725)* putative null mutation [*puf-8(−/−)*] in *GFP:gld-2* 3′ UTR transgenic worms. GFP expression in the germline was also visualized by staining dissected adult hermaphrodite gonads with an anti-GFP polyclonal antibody and DAPI. The expression levels were quantified using ImageJ software. Interestingly, the *puf-8(−/−)* mutant germlines siginifcantly accumulated GFP expression in the distal mitotic germ cells (Figures 3F,G). This difference was particularly striking within the distal mitotic germ cells, where GFP was about ∼25-fold higher in *puf-8(−/−)* mutants than in wild-type worms [*puf-8(+/+)*]. These data suggest that PUF-8 represses *gld-2* mRNA expression via its 3′ UTR in the distal mitotic germ cells.

### *gld-2* Hemizygosity Promotes Germline Tumors in the Absence of PUF-8

**FIGURE 4:**
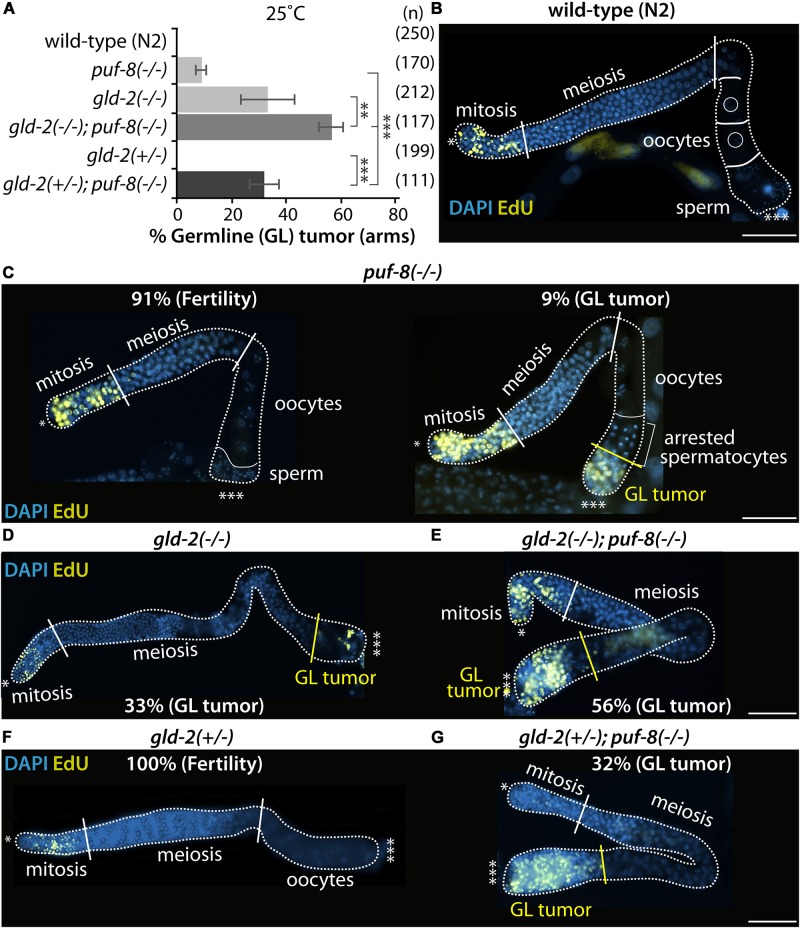
PUF-8 and GLD-2 inhibit the formation of germline tumor. **(A)** The percentage of germline tumors at 25°C. The germline phenotypes were analyzed at 4 days past L1 stage. **(B–G)** Staining of dissected adult hermaphrodite germlines. All were stained using EdU-labeling kit (a marker for mitotic cells) and DAPI (a marker for DNA). ^∗^Distal end of gonads; ^∗∗∗^proximal end of gonads; white broken lines, the boundary of gonad; white lines, the boundary of different germ cell types: mitotic region and meiotic region, meiotic region and oocytes, oocytes and sperm; yellow lines, the boundary between differentiated cells and dedifferentiated EdU-positive mitotic cells. Scale bars, 50 μm.

To assess the biological function of GLD-2 in the formation of *puf-8(−/−)* proximal germline tumors, we examined their germline phenotypes by staining dissected gonads with an EdU-labeling kit (a marker for mitotic cells) and DAPI. The *puf-8(−/−)* homozygote mutants exhibit distinct phenotype at different temperatures. At permissive temperature (20°C), most *puf-8(−/−)* mutants make both sperm and oocytes, resembling wild-type germline (Subramaniam and Seydoux, 2003; Cha et al., 2012). However, at restrictive temperature (25°C), about 9% of *puf-8(−/−)* hermaphrodite mutants (*n* = 170) at 4 days past L1 stage had proximal germline tumors (Figures 4A–C). The germline tumor is an ectopic mass of proliferative mitotic germ cells, which occupy the proximal portion of the adult gonad, a region normally occupied by fully formed gametes. Most *gld-2(q497)* homozygote mutants [*gld-2(−/−)*] make both sperm and oocytes although both gametes are defective at 20°C (Kadyk and Kimble, 1998). However, about 33% of *gld-2(−/−)* hermaphrodite mutants (*n* = 212) exhibit proximal germline tumors by an ill-defined mechanism at 25°C (Figures 4A,D). Notably, the percentage of *puf-8(−/−)* proximal germline tumors was significantly increased by loss of GLD-2 (56%, *n* = 117) (Figures 4A,E). This result indicates that PUF-8 and its repressing target, GLD-2, genetically work together to inhibit the formation of proximal germline tumors. This homeostatic negative-positive regulation of cell fate decision has recently been recognized in invertebrates and extended to vertebrates (Whelan et al., 2012; Datla et al., 2014).

It widely accepted that cell fate can be regulated by gene dosage and genetic context (Thompson et al., 2005; Snow et al., 2013). The potential effect of gene dosage on cell fate was therefore examined in *gld-2(*+/−*); puf-8(−/−)* and *gld-2(−/−); puf-8(*+/−*)* mutants. Most single heterozygote mutants (+/−) for *puf-8* or *gld-2* were normal and did not induce the formation of germline tumors (Figures 4A,F). However, heterozygous mutation for *gld-2* [*gld-2(*+/−*)*] significantly increased the percentage of *puf-8(−/−)* mutants with germline tumors at 25°C (32%, *n* = 111; Figures 4A,G). Notably, the percentage of *gld-2(*+/−*); puf-8(−/−)* exhibiting germline tumors gradually increased during aging (63%, *n* = 162, 6 days past L1 stage). These findings suggest two conclusions: (1) *gld-2* gene is haplo-insufficient in the absence of PUF-8 and (2) *gld-2(*+/−*)* mutation interferes with the differentiation of germlines and promotes the formation of germline tumors in the absence of PUF-8.

### *gld-1* Hemizygosity Further Enhances *gld-2(*+/−*); puf-8(−/−)* Germline Tumors

**FIGURE 5:**
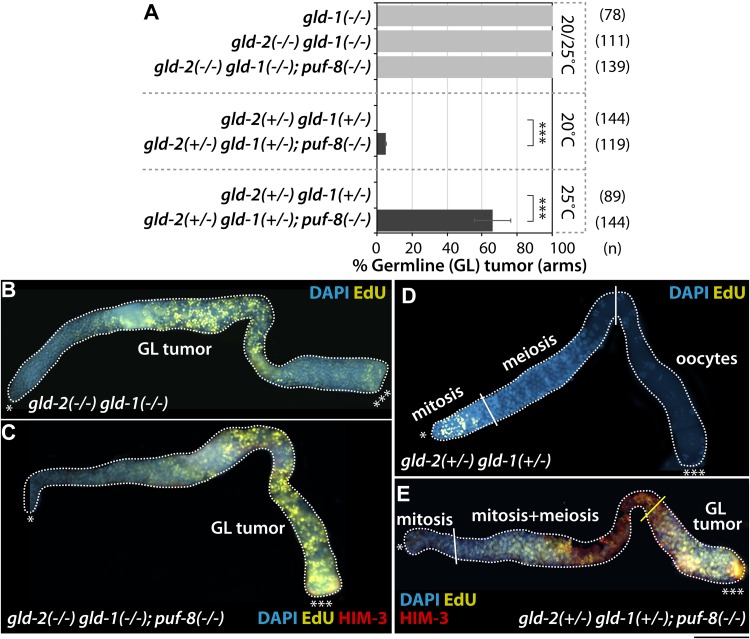
One dose of wild-type *gld-1* gene [*gld-1(*+/−*)*] promotes the formation of *gld-2(*+/−*); puf-8(–/–)* germline tumors. **(A)** The percentage of germline tumors at 20 and 25°C. The germline phenotypes were analyzed at 4 days past L1 stages. **(B–E)** Staining of dissected adult hermaphrodite germlines. All were stained using EdU-labeling kit (a marker for mitotic cells), HIM-3 (a maker for meiotic cells) and DAPI (a marker for DNA). ^∗^Distal end of gonads; ^∗∗∗^proximal end of gonads; white broken lines, the boundary of gonad; white lines, the boundary of different germ cell types: mitotic region and meiotic region, meiotic region and oocytes; yellow lines, the boundary between differentiated cells and dedifferentiated EdU-positive mitotic cells. Scale bars, 50 μm.

It was previously reported that GLD-2 enhances entry into the meiotic cell cycle at least in part by activating *gld-1* mRNA expression (Suh et al., 2006). The *gld-1* gene encodes a STAR/Quaking translational repressor and promotes entry into the meiotic cell cycle (Francis et al., 1995). Thus, *gld-1(q485)* hermaphrodite homozygous mutants [*gld-1(−/−)*] exhibit germline tumors from the female germ cells unable to progress through pachytene in the absence of GLD-1, returning to mitosis (Francis et al., 1995; Jones and Schedl, 1995). To test if one dose of wild-type *gld-1* gene [*gld-1(*+/−*)*] could further enhance the formation of *gld-2(−/−); puf-8(−/−)* and *gld-2(*+/−*); puf-8(−/−)* germline tumors at 25°C, we employed *gld-2(−/−) gld-1(−/−); puf-8(−/−)* and *gld-2(+/−) gld-1(+/−)*; *puf-8(−/−)* triple mutants. GLD-1 levels in heterozygous mutants were quantified with ImageJ software followed by germline staining with anti-GLD-1 (Supplementary Figure S4). Most *gld-2(−/−) gld-1(−/−)*; *puf-8(−/−)* mutants generated germline tumors at both 20 and 25°C as seen in *gld-2(−/−) gld-1(−/−)* germline (Hansen and Schedl, 2006; Figures 5A–C). We also analyzed the germline phenotypes of *gld-2(*+/−*) gld-1(*+/−*)* and *gld-2(*+/−*) gld-1(*+/−*); puf-8(−/−)* mutants grown at 20 and 25°C. The *gld-2(*+/−*) gld-1(*+/−*)* mutants did not form germline tumors at both 20 and 25°C (Figures 5A,D), but 5% (*n* = 119) and 66% (*n* = 144) of *gld-2(+/−) gld-1(+/−)*; *puf-8(−/−)* mutants had germline tumors at 20 and 25°C, respectively (Figures 5A,E). These results indicate that heterozygous *gld-2* and *gld-1* mutations are competent for meiotic entry, but haplo-insufficient for the terminal differentiation of spermatocytes in the absence of PUF-8. Recent evidence indicates that heterozygous deletions are often associated with serious human disease, often called the haplo-insufficient effect, most notably myelodysplastic syndrome and acute myeloid leukemia (Chen et al., 2014). Since these regulators are broadly conserved in humans, our results may be of significant relevance to haplo-insufficiency-associated human disease and underlying molecular mechanisms.

### MPK-1/ERK Is Required for the Formation of *gld-2(*+/−*) gld-1(*+/−*); puf-8(−/−)* Germline Tumors

**FIGURE 6:**
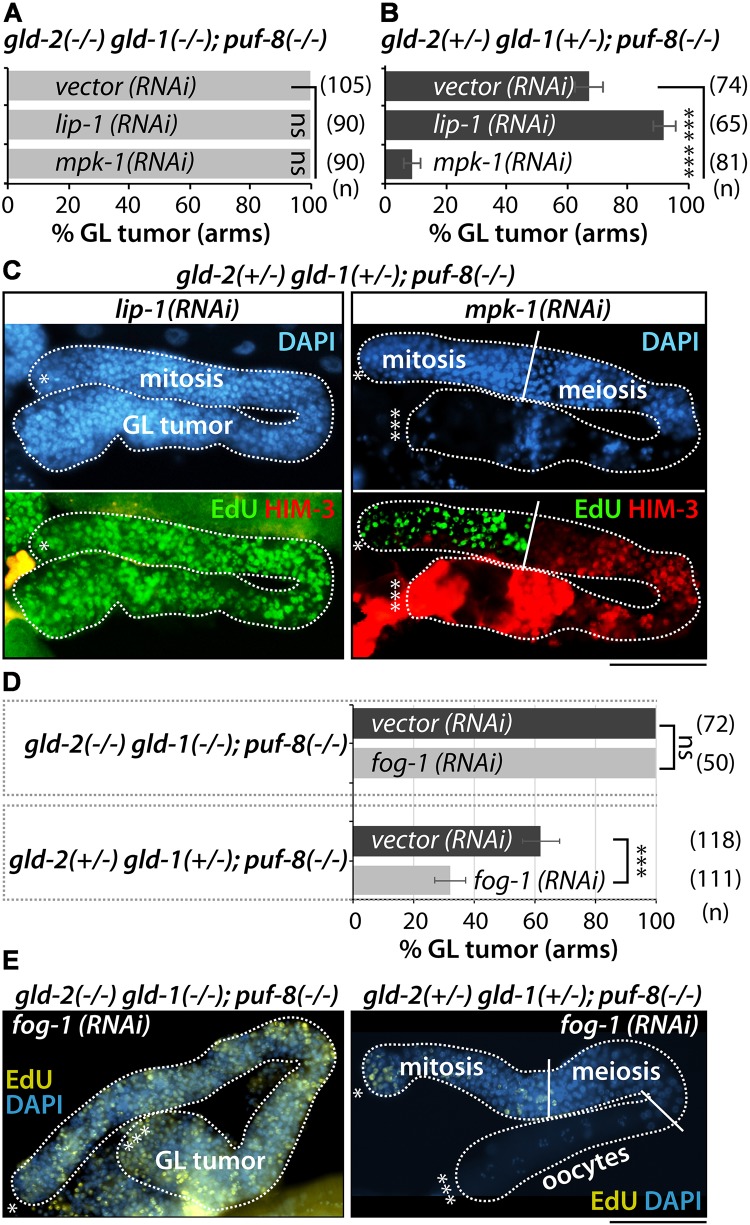
MPK-1 and sperm fate are required for *gld-2(*+/−*) gld-1(*+/−*); puf-8(–/–)* germline tumors. **(A,B)** The percentage of germline tumors at 23°C. The germline phenotypes were analyzed at 4 days past L1 stage. **(C)** Staining of dissected adult hermaphrodite germlines with EdU-labeling kit (a marker for mitotic cells), anti-HIM-3 (a marker for meiotic cells), and DAPI (a marker for DNA). **(D)** The percentage of germline tumors at 25°C. **(E)** Staining of dissected adult hermaphrodite germlines with EdU-labeling kit and DAPI. ^∗^Distal end of gonads; ^∗∗∗^proximal end of gonads; white broken lines, the boundary of gonad; white lines, the boundary of different germ cell types: mitotic region and meiotic region, meiotic region and oocytes. Scale bars, 50 μm.

We previously reported that the activation of MPK-1 by loss of LIP-1 (a dual specificity phosphatase) in the absence of PUF-8 initiates the formation of germline tumors as spermatocytes revert back into mitotic cells via a dedifferentiation-like mechanism (Cha et al., 2012). To examine the dependence of *gld-2(−/−) gld-1(−/−); puf-8(−/−)* and *gld-2(*+/−*) gld-1(+/−*);* puf-8(−/−)* germline tumor formation on MPK-1 activity, we performed *lip-1(RNAi)* (MPK-1 activation) or *mpk-1(RNAi)* (MPK-1 inhibition) on synchronized L1 larvae at a moderate temperature (23°C). Their germline phenotypes were analyzed by staining dissected gonads with EdU-labeling kit and anti-HIM-3 (a marker for meiotic cells) antibody at adult stage (4 days past L1). No *gld-2(−/−) gld-1(−/−); puf-8(−/−)* germline tumors were affected by *lip-1(RNAi)* or *mpk-1(RNAi)* (Figure 6A). However, *gld-2(*+/−*) gld-1(*+/−*); puf-8(−/−)* germline tumors were significantly enhanced by *lip-1(RNAi)* (*p* < 0.0017, *n* = 65) and inhibited by *mpk-1(RNAi)* (*p* < 0.0001, *n* = 81) (Figures 6B,C). Similarly, the germline tumors of *gld-2(*+/−*); puf-8(−/−)* mutants were also suppressed by *mpk-1(RNAi)* (Supplementary Figure S5). These findings suggest two conclusions: (1) MPK-1 is critical for the formation of *gld-2(*+/−*) gld-1(*+/−*); puf-8(−/−)* germline tumors at 25°C, and (2) the mechanisms of *gld-2(−/−) gld-1(−/−); puf-8(−/−)* and *gld-2(*+/−*) gld-1(*+/−*); puf-8(−/−)* germline tumor formation are different.

### *gld-2(*+/−*) gld-1(*+/−*); puf-8(−/−)* Germline Tumors Arise From Spermatocytes via Dedifferentiation-Like Mechanism

MPK-1 is required for pachytene exit (Lee et al., 2007). Since loss of MPK-1 arrests germ cells in pachytene, resulting in no sperm and oocytes (Lee et al., 2007), we speculate that the germline tumor of *gld-2(*+/−*) gld-1(*+/−*); puf-8(−/−)* germline tumors may arise after pachytene exit. Importantly, previous studies by us and Dr. Seydoux’ group revealed that *puf-8(−/−)* germline tumors arise from the return of spermatocytes into mitotic cells (Subramaniam and Seydoux, 2003; Cha et al., 2012). To test if the germline tumors of *gld-2(*+/−*)* gld-1(+/−*); puf-8(−/−)* mutants, but not *gld-2(−/−) gld-1(−/−); puf-8(−/−)* mutants, are similarly derived from spermatocytes, we blocked sperm fate specification by *fog-1(RNAi)*. *fog-1* encodes cytoplasmic polyadenylation element binding (CPEB) protein and is critical for sperm fate specification (Barton and Kimble, 1990). *fog-1(RNAi)* inhibits sperm fate and instead promotes oocyte specification. *fog-1(RNAi)* was started in synchronized L1 larvae at 25°C and germline phenotypes were analyzed 4 days past L1 stage by staining dissected gonads with an EdU labeling kit and DAPI. The efficiency of *fog-1(RNAi)* was confirmed in wild-type (N2) worms. About 90% of wild-type worms were feminized by *fog-1(RNAi)* at 25°C (data not shown). Of note, *fog-1(RNAi)* significantly suppressed the germline tumors of *gld-2(*+/−*)* gld-1(+/−*); puf-8(−/−)* mutants, but not those of *gld-2(−/−) gld-1(−/−); puf-8(−/−)* mutants at 25°C (Figures 6D,E). Similarly, the germline tumors of *gld-2(*+/−*); puf-8(−/−)* mutants were also suppressed by *fog-1(RNAi)* (Supplementary Figure S5). These results suggest two conclusions: (1) *gld-2(−/−) gld-1(−/−); puf-8(−/−)* germline tumors are derived from the failure of meiotic entry in the distal mitotic cells as seen in *gld-2(−/−) gld-1(−/−)* germline tumors, and (2) conversely, *gld-2(*+/−*) gld-1(*+/−*); puf-8(−/−)* germline tumors may arise from spermatogenic germ cells via dedifferentiation-like mechanism in the proximal germ cells.

## Discussion

**FIGURE 7:**
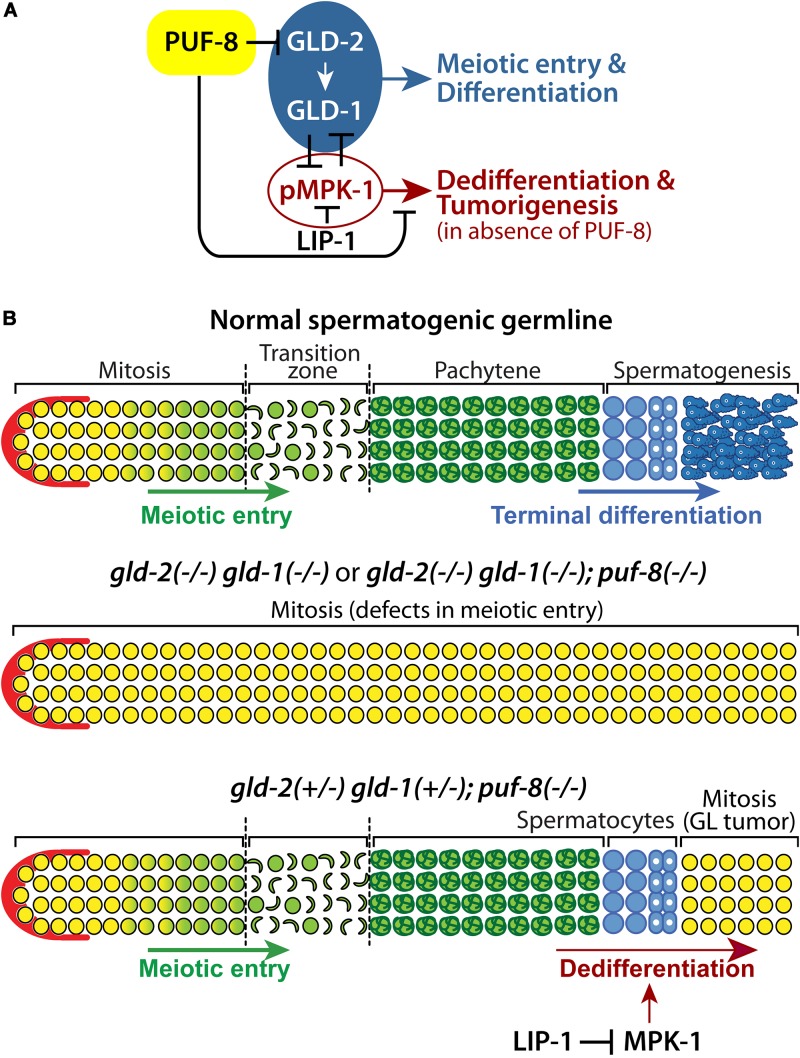
A proposed regulatory network that controls germline differentiation, dedifferentiation, and tumorigenesis. **(A)** PUF-8 represses both germline differentiation and dedifferentiation (and tumorigenesis) by inhibiting GLD-2 and MPK-1 signaling. **(B)** In a normal spermatogenic germline (top), increased GLD-2 and GLD-1 promote germline differentiation. In *gld-2(–/–) gld-1(–/–)* or *gld-2(–/–) gld-1(–/–); puf-8(–/–)* mutant germlines (middle), germ cells fail to enter meiotic cell cycle and continue to proliferate, resulting in germline tumors. In *gld-2(*+/−*) gld-1(*+/−*); puf-8(–/–)* mutant germline (bottom), germ cells enter meiotic cell cycle, but spermatocytes return into mitotic cell cycle through dedifferentiation-like mechanism at 25°C. Dedifferentiation-mediated germline tumors depend on MPK-1 activity.

Differentiation programs of stem cells depend on gene expression largely regulated at the level of mRNAs. Recently, mRNA regulation has emerged as one of the key mechanisms that control the differentiation of stem cells into terminal cell types during animal development (Shigunov and Dallagiovanna, 2015). In this study, we have identified *gld-2* as a PUF-8 target mRNA. Our genetic analyses demonstrated that PUF-8 and its repressing target GLD-2 promote germline differentiation by activating GLD-1 and inhibiting MPK-1 in the *C. elegans* germline (Figure 7). However, one dose of wild-type *gld-2* and *gld-1* genes [*gld-2(*+/−*) gld-1(*+/−*)*] in the absence of PUF-8 is insufficient for the terminal differentiation of spermatocytes, and instead promotes the formation of germline tumors via a dedifferentiation-like mechanism by activating MPK-1 (Figure 7). These findings suggest that a regulatory circuit involving PUF-8, GLD-1, GLD-2, and MPK-1 controls the program of germline differentiation or dedifferentiation depending on gene dosage and genetic context (Figure 7).

### Gene Dosage Effects on Germ Cell Fate Specification

Among *C. elegans* PUF proteins, PUF-8 is the most similar to human PUMILIO (Wickens et al., 2002; Datla et al., 2014). PUF-8 controls multiple cellular processes during germline development, depending on genetic context (Datla et al., 2014). During early germline development, PUF-8 and MEX-3 (a KH domain translational regulator) contribute to the maintenance of GSCs by promoting mitotic proliferation (Ariz et al., 2009). However, PUF-8 also inhibits the proliferative fate of germ cells by inhibiting GLP-1/Notch signaling or by functioning parallel to it (Racher and Hansen, 2012). Once germ cells enter meiotic cell cycle, PUF-8 works together with LIP-1 to promote oocyte fate at the expense of sperm fate by repressing MPK-1 activation at permissive temperature (20°C) (Subramaniam and Seydoux, 2003; Cha et al., 2012). Notably, PUF-8 and LIP-1 also inhibit the formation of germline tumors by promoting the meiotic completion of spermatocytes at restrictive temperature (25°C). In spite of the well documented diverse functions of PUF-8, only a few PUF-8 targets have been identified to date (Mainpal et al., 2011). In the current study, *in silico* and biochemical analyses have identified *gld-2* as a *bona fide* direct PUF-8 target mRNA (Figure 2). PUF-8 and GLD-2 have opposite biochemical and biological functions. While PUF-8 inhibits mRNA translation, GLD-2 activates it. GLD-2 also plays multiple roles by interacting with distinct RNA-binding protein partners; namely GLD-3 and RNP-8 (an RRM RNA binding protein). GLD-2-GLD-3 and GLD-2-RNP-8 exist as separate cytoplasmic poly(A) polymerase complexes, and they appear to have distinct RNA-binding specificities (Kim et al., 2010). Functionally, GLD-2-GLD-3 complex promotes meiotic entry and sperm fate, whereas GLD-2-RNP-8 complex specifies an oocyte fate (Kim et al., 2009). In particular, GLD-2-GLD-3 complex promotes meiotic entry by activating the translation of *gld-1* mRNAs in the *C. elegans* germline (Suh et al., 2006). This report demonstrates that while one dose of wild-type *gld-2* and *gld-1* genes [*gld-2(*+/−*) gld-1(*+/−*)*] promotes meiotic entry, it nevertheless remains insufficient for the meiotic completion of spermatocytes in the absence of PUF-8. This eventually causes spermatocytes to revert back into mitotic cells by activating MPK-1, resulting in germline tumors (Figure 7B). These RNA regulators (PUF-8, GLD-1, and GLD-2) play critical roles in RNA stability and its translation of numerous genes that are involved in key developmental and cellular processes. In addition, a precise interplay between these regulators at normal expressional levels also establishes a regulatory network for germ cell fate specification and homeostasis. For example, *C. elegans* FOG-1 is critical for sperm fate specification in the germline (Barton and Kimble, 1990). However, low FOG-1 levels [*fog-1(*+/−*)*] promote germline proliferation in the absence of FBF-1 and FBF-2 (*C. elegans* PUF proteins) (Thompson et al., 2005). Likewise, *C. elegans* FOG-3 (a homolog of vertebrate TOB/BTG) is also vital for sperm fate specification, but it can either promote or inhibit germline proliferation in a manner that is sensitive to both genetic context and gene dosage (Snow et al., 2013). Since the effects of gene dosage and genetic context on cell fate specification have recently emerged in vertebrate systems (Stefanovic and Puceat, 2007; Wang et al., 2010; Deo et al., 2013; Bankaitis et al., 2018), we thus suggest that gene dose-dependent control of cell fates may be conserved from worms to mammals, including humans.

### Gradient-Mediated Cell Fate Decision *in vivo*

How are germ cell fates determined depending on dosage and genetic context? While it still eludes us, a suggested gradient model for cell fate decision is presented. Germ cell fates may be governed by relative levels of key regulators at a certain time and place. For example, at the distal end of the gonad, a somatic distal tip cell (DTC) provides a GSC niche and signals to the GSCs via the Notch signaling pathway. Notch signaling activates the transcription of target mRNAs, which are highly expressed in the mitotic cells but not in the meiotic cells. Well studied genes include *sygl-1* and *lst-1* (Kershner et al., 2014). These regulators work together with others (e.g., RNA regulators and cell cycle regulators) to build a regulatory network for mitotic cell fate. As the expression of these regulators is suppressed by a regulatory network for meiotic entry (early differentiation), germ cells enter meiotic cell cycle and their cell fates are maintained during differentiation. Importantly, regulators for mitosis and meiosis antagonize each other and generate an overlap area that may be critical for the mitosis-meiosis decision. Germ cells at meiotic cell cycle are then required to make the sperm-oocyte decision. Notably, many regulators for the mitosis-meiosis decision also play critical roles in the sperm-oocyte decision, including FBFs, PUF-8, GLDs, NOS-3, FOGs, and MPK-1 (Kimble and Crittenden, 2002; Morgan et al., 2013). This finding suggests that a relative level (or ratio) of these regulators at a particular time and place may determine sperm or oocyte fate. In this study, we demonstrated that germ cells also decide whether to remain in a meiotic differentiating state or revert to being undifferentiated. Two key players, PUF-8 and GLD-1, regulate this decision with distinct mechanisms. In oogenic germline, GLD-1 is required for the maintenance of meiotic cell fate by regulating its target mRNAs. Thus, in the *gld-1(−/−)* oogenic germline, early meiotic cells return into mitotic cell cycle, resulting in germline tumors (Jones and Schedl, 1995). In contrast, PUF-8 inhibits the regression of spermatogenic germ cells into mitotically dividing cells only in spermatogenic germline (Subramaniam and Seydoux, 2003; Cha et al., 2012). It was previously reported that PUF-8 functions redundantly with GLD-1 to promote the meiotic progression of spermatocytes in *C. elegans* germline (Priti and Subramaniam, 2015). These results indicate that the expressional levels of PUF-8 and GLD-1 may govern the decision between the maintenance of the meiotic differentiating state and the regression to the mitotic undifferentiating state (also known as the differentiation-dedifferentiation decision). We here demonstrate that one dose of wild-type *gld-2* and *gld-1* genes disrupt the frame of the differentiation-dedifferentiation decision in the absence of PUF-8 (Figure 7). Although it still eludes us what ratio of these regulators influence decisions at a particular time and place, our data show that one dose of wild-type *gld-2* and *gld-1* genes [*gld-2(*+/−*) gld-1(*+/−*)*] are competent for meiotic entry, but are insufficient for the terminal differentiation of spermatocytes in the absence of PUF-8, resulting in dedifferentiation-mediated germline tumors (Figure 7). Notably, the *Drosophila* blastoderm and the vertebrate neural tube are typical examples of gradient-mediated cell fate decision spatially (Briscoe and Small, 2015). In both tissues, cell fate decision relies on molecular gradients. First, signaling gradients establish initial conditions. Second, these signaling gradients initiate transcriptional networks, including activators and repressors, to generate patterns of gene expression. Third, the precise positioning of boundaries temporally and spatially determines commitment to specific cell types. Similarly, regulation of mammalian stem cell proliferation and cell fate decision relies on gradients of signaling molecules and an interplay between activators and repressors in specific tissue compartment boundaries (Du et al., 2015; Tian et al., 2016). This suggests that gradient-mediated cell fate decision may be an evolutionarily conserved mechanism from worms to humans. Collectively, our findings from the simple worm model may provide a novel insight into gradient (and/or gene dose)-mediated cell fate decision in mammals, where such *in vivo* methods are not yet feasible or practical.

## Materials and Methods

### Nematode Strains

All strains were derived from Bristol strain N2 and maintained at 20°C as described unless otherwise noted (Brenner, 1974). Mutations and balancers used in this work include: *LG I*: *gld-2(q497)*, *gld-1(q485)*, *hT2 [bli-4(e937) let-?(q782) qIs48]*, *LG II*: *puf-8(q725)*, *mIn1[mIs14 dpy-10(e128)], IT31 (puf-8:gfp)* (Ariz et al., 2009), *IT689 (GFP:gld-2 3′ UTR)*, and *puf-8(q725); IT689 (GFP:gld-2 3′ UTR).*

### Feeding RNA Interference (RNAi)

RNA interference experiments were performed by feeding bacteria expressing double-stranded RNAs corresponding to the gene of interest (Kamath et al., 2001; Ashrafi et al., 2003). RNAi bacteria were from *C. elegans* ORF-RNAi library (Source BioScience, Nottingham, United Kingdom). Synchronized L1 staged worms were placed on RNAi plates (a NGM plate containing 100 μg/mL Ampicillin and 0.5 mM IPTG) and incubated for 4 days at 20 or 25°C.

### Germline Antibody Staining

Germline antibody staining was performed as previously described (Yoon et al., 2016). Briefly, dissected gonads were fixed in 3% paraformaldehyde/0.1M K_2_HPO_4_ (pH 7.2) solution for 10–20 min, and then post-fixed with cold 100% methanol for 5 min. After blocking for 30 min in 1× PBST (1XPBS + 0.1% Tween 20)/0.5% BSA (Bovine Serum Albumin) solution, primary antibody was added, followed by incubation for 2 h at 20°C (or overnight at 4°C). The dissected gonads were washed three times for at least 30 min with 1× PBST/0.5% BSA solution and incubated in the same solution containing the fluorescence-conjugated secondary antibodies for 1–2 h at 20°C. After washing three times in 1× PBST/0.5% BSA solution for at least 30 min, the dissected gonads were stained with 100 ng/mL DAPI solution for 10 min at 20°C and were again washed in 1× PBST/0.5% BSA solution three times. The antibody staining was observed using fluorescence microscopy. Primary antibodies used in this study include anti-HIM-3 (Novus Biologicals, 1:400 dilution), anti-GLD-1 (provided by Dr. Kimble’s Lab, 1:200 dilution), anti-MSP (Developmental Studies Hybridoma Bank – University of Iowa), and anti-GFP (Abcam, Cambridge, MA, United States, 1:400 dilution). Alexa 488- or CY3-conjugated secondary antibodies (Thermo Fisher Scientific, Waltham, MA, United States, 1:200 dilution) were diluted to 1:300.

### EdU (5-Ethynyl-2′-Deoxyuridine) Labeling

To label mitotically cycling cells, worms were incubated with rocking in 0.2 mL M9 buffer (3 g KH_2_PO_4_, 6 g Na_2_HPO_4_, 5 g NaCl, 1 mL 1M MgSO_4_, H_2_O to 1 L) containing 0.1% Tween 20 and 1 mM EdU for 30 min at 20°C. Gonads were dissected and fixed in 3% paraformaldehyde/0.1M K_2_HPO_4_ (pH 7.2) solution for 10–20 min, followed by −20°C methanol fixation for 10 min. Fixed gonads were blocked in 1× PBST/0.5% BSA solution for 30 min at 20°C. EdU labeling was performed using the Click-iT EdU Alexa Fluor 488 Imaging Kit (Invitrogen, CA, United States, #C10337), according to the manufacturer’s instructions. For co-staining with antibodies, EdU-labeled gonads were incubated in the primary antibodies after washing for three times, and subsequently in the secondary antibodies as described above.

### Yeast Three-Hybrid, 3-AT, and Gel Retardation Assays

Three-hybrid assays were performed as previously described (Hook et al., 2005). The sequences for the 3′ UTR region of *gld-2* were cloned using the pIIIa/MS2-2 vector (provided by Dr. Wickens, University of Wisconsin–Madison). These vectors, containing the target sequences of *gld-2* 3′ UTR, were co-transformed with PUF-8:pACTII vector into YBZ-1 yeast strain. The level of β-galatactosidase was assayed in at least three independent experiments. The strength of PUF protein-RNA interaction was determined by the 3-AT assay. The levels of 3-AT resistance were measured by assaying multiple transformants at four different concentrations of 3-AT, from 1 to 10 mM. Gel retardation assays were performed as previously described (Hook et al., 2005).

### Data Analysis

Statistical significance was analyzed using one-way analysis of variance (ANOVA). The error bars reflect respective standard deviation values. ^∗^*p* < 0.05, ^∗∗^*p* < 0.01, ^∗∗∗^*p* < 0.001.

## Data Availability Statement

This manuscript contains previously unpublished data. The name of the repository and accession number(s) are not available.

## Author Contributions

YP, SO, FT, and ML performed the experiments. All authors contributed the reagents, materials, and analysis tools. ML and MA designed the experiments, analyzed the data, and wrote the manuscript.

## Conflict of Interest

The authors declare that the research was conducted in the absence of any commercial or financial relationships that could be construed as a potential conflict of interest.
