# Supplementary material for: Dose-Dependent Effects of GLD-2 and GLD-1 on Germline Differentiation and Dedifferentiation in the Absence of PUF-8
Source: Front Cell Dev Biol. 2020 Jan 24;8:5. doi: 10.3389/fcell.2020.00005 (PMC6992537; doi:10.3389/fcell.2020.00005)
Supplement: Supplementary file 1 [file Data_Sheet_1.PDF]

Supplementary Material for

Dose-Dependent Effects of GLD-2 and GLD-1 on Germline Differentiation and Dedifferentiation in the Absence of PUF-8

Youngyong Park[a], Sam O'Rourke[a], Faten A. Taki[b], Mohammad A. Alfhili[a,c], Myon Hee Lee[a,#]

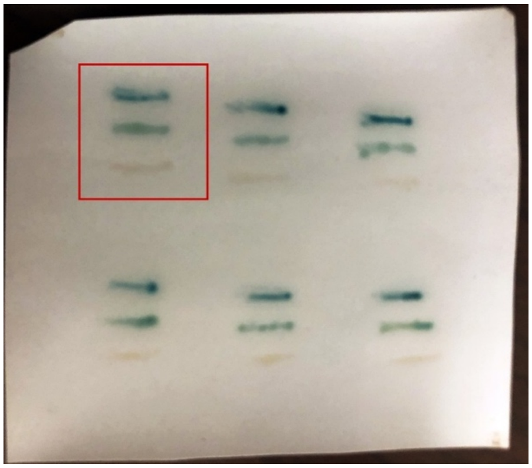

Figure S1. Original Blot for Figure 2C

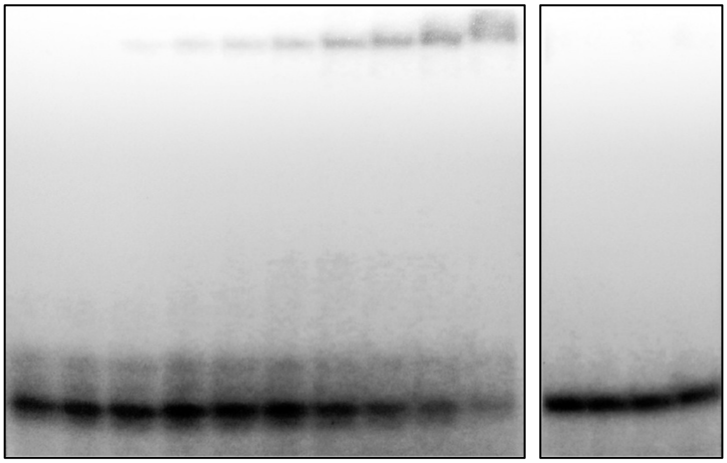

Figure S2. Original Blot for Figure 2E

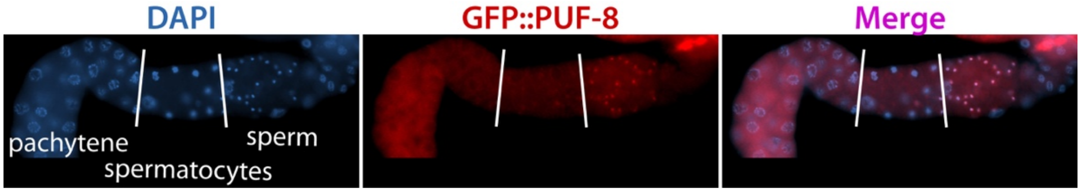

Figure S3. The expression of GFP::PUF-8 in L4 staged spermatogenic hermaphrodite germline. Antibody staining of dissected gonads with anti-GFP antibody.

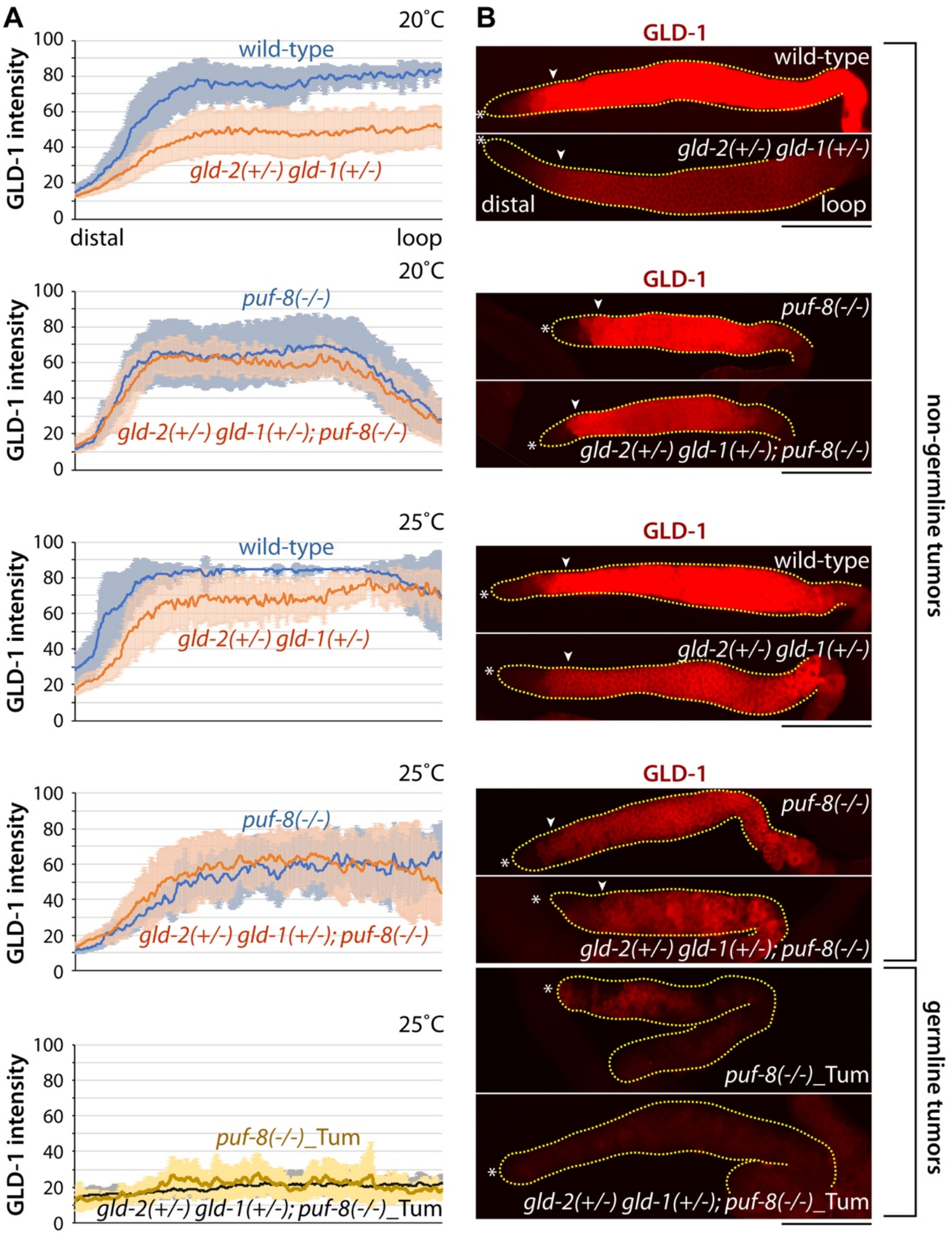

Figure S4. The levels of GLD-1 protein. (A) Quantitation of GLD-1 protein. The intensity of GLD-1 protein was quantified using ImageJ software. The x-axis represents distance from distal tip (*) of the germline, and the y-axis is pixel intensity. Error bars, standard deviation. (B) Antibody staining dissected adult hermaphrodite germlines with anti-GLD-1. Scale bars, 50 μm.

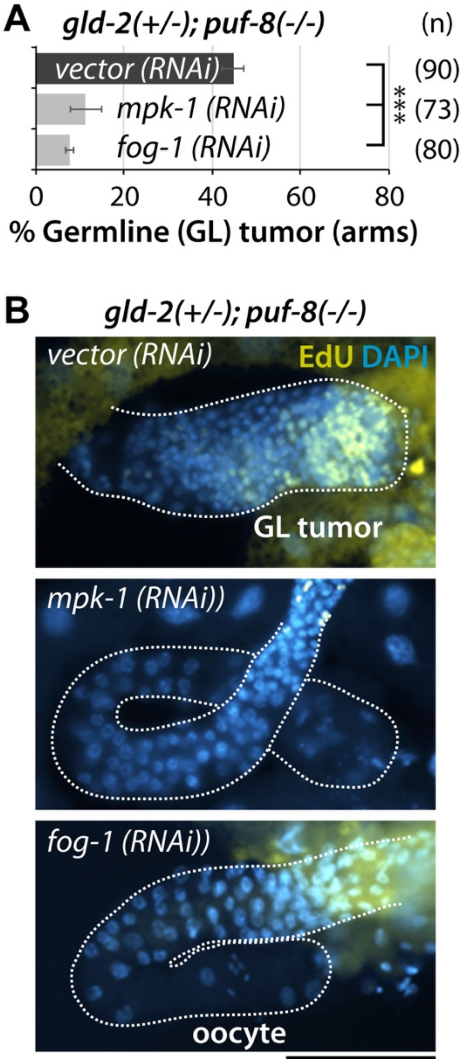

Figure S5. MPK-1 and sperm fate are required for *gld-2(+/-); puf-8(-/-)* germline (GL) tumors. (A) The percentage of germline tumors at 25°C. The germline phenotypes were analyzed at 4 days past L1 stage. (B) Staining of dissected adult hermaphrodite germlines with EdU-labelling kit and DAPI. Scale bars, 50 μm
